# From not knowing, to knowing more needs to be done: health care providers describe the education they need to care for sex trafficked patients

**DOI:** 10.1186/s12909-024-05776-6

**Published:** 2024-07-31

**Authors:** Robin Mason, Frances Recknor, Rhonelle Bruder, Fareeha Quayyum, Frances Montemurro, Janice Du Mont

**Affiliations:** 1 Women’s College Research Institute, Women’s College Hospital, 76 Grenville St, Toronto, ON M5S 1B2 Canada; 2 Dalla Lana School of Public Health, University of Toronto, 155 College St, Toronto, ON M5T 3M7 Canada

**Keywords:** Education, Qualitative, Sex trafficking, Training

## Abstract

**Background:**

Sex trafficking is highly prevalent, pernicious, and under-recognized. When an individual is trafficked for the purpose of sexual exploitation within the borders of a single country, it is termed domestic sex trafficking. Sex trafficked persons can experience severe physical and mental health outcomes requiring medical attention and treatment. However, health care providers often fail to identify sex trafficked patients, missing opportunities to provide needed care and support.

**Methods:**

In this qualitative study, we interviewed 31 health care providers (physicians, nurses, and social workers) working in Ontario, Canada to learn what they identified as their specific education and training needs to recognize and care for sex trafficked persons. Interviews were conducted over Zoom, recorded, and transcribed. Coding of the transcripts followed a standard framework for qualitative studies. Codes related to the education and training needs of providers were identified as a core issue suited to further analysis.

**Results:**

Three themes related to providers’ education and training needs emerged. These acknowledge basic (*Foundational knowledge)*, as well as more specific learning needs (*Navigating the encounter)*. The final theme, (“*It just seems so much bigger than me”)* suggests that even with some knowledge of domestic sex trafficking, participants still experienced considerable distress and multiple challenges due to gaps in the broader system impacting the provision of appropriate care.

**Conclusions:**

Participants voiced their need for specialized sex trafficking education as well as role specific training to combat their sense of inadequacy and provide better care for their patients. Participants’ education needs ranged from requiring the definition of domestic sex trafficking and the frequency of its occurrence, to the various circumstances associated with increased risk of recruitment into sex trafficking. In terms of desired training and specific skills, participants wanted to learn how to identify a person being sex trafficked, broach the subject with a patient, know what to do next including access to local resources and referrals, as well as connections to other critical services, such as legal and housing. The results can be used to inform the design and content of education and training on sex trafficking for health care providers.

## Background

Human trafficking is the “very antithesis of social justice” [1]. Involving the recruitment, movement, or holding of persons for the purpose of exploitation, sex trafficking is a particular form of human trafficking wherein individuals are used for sexual services. Sex trafficking that does not cross international borders but originates and occurs within the borders of one country is termed domestic sex trafficking [2]. Unduly affecting historically marginalized persons, and with precise estimates difficult to discern, the International Labour Organization estimates trafficking affects 50 million people globally, with 6.3 million persons exploited sexually [1]. Women and girls are the identified victims in the majority of police reported incidents of human trafficking in Canada. Ontario, the country’s most densely inhabited province, stands out as a key site of domestic sex trafficking [3, 4]. As traffickers are known to use a range of physical and psychological tactics to control their victims [5], it is not surprising that there are physical and mental health repercussions for trafficked persons and that these can last long after they have left their trafficked circumstances [5, 6]. 

### The intersection of health, health care, and sex trafficking

Zimmerman et al.’s [7] multi-country 2008 study surveyed 192 women receiving post-trafficking services. In the seven days prior to participation, 63% reported a minimum of 10 coincident physical health problems, high rates of depression and anxiety, and 39% expressed suicidal thoughts [7]. More recent studies have further detailed the many profound impacts of trafficking on the reproductive, physical, and mental health of trafficked persons [8, 9]. Sexually transmitted infections, Hepatitis C, pelvic pain, complications of pregnancy, and forced abortions are some of the reproductive issues experienced. Other notable physical illness and injuries included neurological symptoms, such as migraines and dizziness; malnutrition and severe weight loss due to inadequate nutrition; dental disease; accidental and intentional injuries, including fractures, and those from being punched, stabbed, kicked, and/or sexually assaulted [8, 9]. Also common were mental health problems related to depression, anxiety, flashbacks, nightmares, and substance use [8, 9]. In one study, 41% of participants attempted suicide while trafficked [8]. 

Given the magnitude and extent of these physical and mental health issues, it is also not surprising that up to nearly 90% of those who have been trafficked access health care either while trafficked or shortly thereafter [8, 10, 11]. Unfortunately, sex trafficked individuals [8] and those trafficked for other labour are, at best, “inconsistently identified” [12 (p.1229)] by health care providers whom, it has been suggested, lack the education and training to recognize key behaviours or other indicators associated with sex trafficking [13]. 

A nascent literature studying health care providers’ identification of sex trafficked persons, particularly in Emergency settings, has emerged [13, 14]. As well, some Canadian studies focused on social service providers’ knowledge of sex trafficking have been conducted  [16–19]. However, to date there has been little focus on Canadian health care providers and what they say they need to know to better care and support domestically sex trafficked persons  [15, 20]. Of the few Canadian studies, one examined Canadian medical students and another residents’ knowledge of human trafficking but neither specified sex trafficking [21, 22]. In the first study, medical students attending the largest medical school in Ontario, Canada, were surveyed about their awareness of and attitudes toward human trafficking. Almost half of those surveyed reported being not knowledgeable, the remainder as somewhat knowledgeable, while nearly all said they were unfamiliar with the signs and symptoms of trafficking [21]. The second study evaluated the impact of a 30–60-minute education session on human trafficking on 32 Canadian medical residents. Pre-intervention, 6% felt somewhat knowledgeable about human trafficking and 16% said they recognized trafficking indicators. Less than a third (31%) had received any previous education about human trafficking but 81% agreed or strongly agreed that it should be integrated into resident education [22]. 

While it is crucial to the future care of sex trafficked persons that medical students and residents receive needed education on sex trafficking, it is equally important to consider the education and training needs of those already working in health care settings. In response to this notable gap, in this study we explored what Ontario physicians, nurses, and social workers said they need to learn to better care for sex trafficked patients. With this information, relevant continuing education initiatives, as well as updates to undergraduate curricula, can be developed and implemented. This in turn, will lead to better care for domestically sex trafficked patients and less anxiety, stress, and confusion for health care providers in Ontario and beyond.

## Method

### Study design

This qualitative study employed semi-structured, one-on-one interviews with health care providers to focus on fleshing out our earlier recommendation on the need for sex trafficking education and guidelines for health care providers [23]. The interview guide was developed as part of a larger program of research exploring different professionals’ knowledge of, and ability to respond to, domestically sex trafficked adolescents and adults in Ontario [15–19]. In developing the interview guide, we drew upon items from Cunningham and DeMarni Cromer’s (2016) Human Trafficking Myths Scale [24], as well as a review of the literature, and team members’ varied expertise [23]. Examples of questions included: How would you identify someone who is being sex trafficked? What guidelines or protocols do you have to follow when caring for a person who has been sex trafficked?” The study, part of the larger research program, was approved by the Women’s College Hospital’s Research Ethics Board in December 2021 (REB# 2021-0133-E) [15–18]. 

### Recruitment and study participants

Purposeful and snowball sampling were used to recruit physicians, nurses, and social workers working in a hospital, community clinic or similar health care setting in the province of Ontario. Recruitment took place over a four-month period between November 2022 and February 2023. Emails explaining the study and recruitment flyers were shared with health care organizations including: community health centres; family practices and health teams; walk-in clinics; women’s health and abortion and birth control clinics; health care provider professional organizations; clinical programs addressing psychiatric, substance use, trauma and generalized mental health issues; and programs delivering health care and mental health services specifically for Indigenous peoples and 2SLGBTQI + populations. Those interested in participating were sent a sociodemographic questionnaire which also served to confirm eligibility. Experience with sex trafficked persons was not a requirement of participation. A consent form describing study aims, potential risks and benefits of participation, steps taken to maintain anonymity and confidentiality, and that interviews would be audio/video recorded was sent to interested parties. Once potential participants had any outstanding questions about the study addressed, the form was signed and returned to the research team.

### Data collection

Two team members conducted the interviews. To enhance consistency in approach and fidelity in use of the interview guide, the two interviewers first practiced interviewing each other while a third team member observed and provided feedback. Once satisfactory consistency was achieved, interviews were scheduled and conducted over Zoom between November 2022 and February 2023. No administrative or other issues arose during the interview phase. At the start of the interview, the participant was asked to again consent to the audio and/or video recording. Interviews lasted between one and two hours. Immediately following the interview, the interviewer recorded their initial reflections and observations, uploaded the transcript and audio/video file to the Women’s College Hospital’s secure online directory for team members only, and deleted the file from Zoom. After checking the transcript for accuracy against the audio recording and removing personal details that might compromise participant privacy (de-identification), transcripts were imported into a password protected software management program, Dedoose Management Software (Version 9.0.90, 2023). Participants selected pseudonyms which were then assigned to each transcript. Participants received an honorarium in the form of a $25 CAD e-gift card.

### Data analysis and rigor

Data analysis followed Braun & Clarke’s six-phase framework [25]. Phase I and II, data familiarization and initial code generation, were iterative processes as four team members independently read four transcripts to first generate deductive codes in response to specific questions, then inductive codes and subcodes. Over a series of meetings, team members compared the codes, resolved the few differences in coding, amalgamated some and deleted others, and developed code definitions. The result was the preliminary codebook. Two additional transcripts were then independently coded by two team members using this preliminary codebook, adding new codes as needed. The team again met to review, refine, and confirm or discard codes and resolve any outstanding differences; this resulted in the final codebook. In Phase III, team members worked on coding the remaining transcripts using the finalized codebook. The codes and subcodes related to health care providers’ education and training needs emerged as a core domain and sufficiently rich to warrant separate consideration during Phase IV. We reviewed, consolidated, and analyzed this set of codes to establish the themes shared in this paper. In Phase V, we created a table with representative quotes to illustrate each theme. We prepared the manuscript as part of Phase VI [26]. 

In addition to the steps noted above, several other measures were taken to enhance rigor and trustworthiness. Following each interview and throughout the coding process, the two interviewers independently noted their thoughts and impressions in a reflexive journal and held regular peer debriefings. Routine meetings of the entire team were held to discuss project progress, emerging themes, lessons learned, and the potential impact of any of our own biases on data analysis. Critical decisions were made collectively by consensus. The record of decisions and meetings, in addition to the reflexive journals, contributed to an audit trail enhancing trustworthiness [26–28]. The Consolidated Criteria for Reporting Qualitative Research guidelines were used in the write-up of study findings and manuscript preparation [29]. 

## Results

Thirty-one interviews were completed and data saturation reached [27, 30]. The participants included physicians (9), nurses (10), and social workers (12). Twenty-six identified as women and five as men. Ages ranged from 25 to 69 years. Eighteen participants identified as White, one identified as a member of First Nations, while others indicated varied races/ethnicities. Twenty-three participants worked in urban areas, three in suburban, two in urban/suburban, two in rural, and one in a remote location. Three providers reported having cared for more than 10 sex trafficked persons/year, two indicated 6–10/year, nine said 1–5/year, four had seen 0, and the remaining 13 said “I don’t know,” or did not answer.

Three core themes emerged from the analysis of what health care providers said were their education and training needs. The themes reflect participants’ aspirations for both basic (*Foundational knowledge)* and the more specific education needed to better care for a sex trafficked patient (*Navigating the encounter)*. The final theme, (*It just seems so much bigger than me)* suggests that even with some knowledge and understanding of domestic sex trafficking, participants still experienced considerable distress and multiple challenges when trying to provide appropriate care. Each of these themes is described with supporting participant quotes. As physicians’, nurses’, and social workers’ roles differ in terms of, for example, responsibilities and scope of practice, the participant’s profession is provided with each quote along with their pseudonym.

### Foundational knowledge

Participants indicated they required general knowledge about domestic sex trafficking including definition, frequency of occurrence, ‘risk’ factors and those most vulnerable, biases and myths, recruitment processes used by predators, and red flags (see Table 1). For example, one physician noted that as with any issue, one needs to first understand “the background and definitions […] [and] then talk about the epidemiology, and the sort of classic presentation […] the typical demographics” (John), while another physician, Sandra Smith, echoed this, suggesting that what was needed was “information about the particular demographics of those that are at risk and, you know, the things to kind of look out for.” Jane, a social worker, agreed saying providers need to know that “it [sex trafficking] occurs, and that it occurs locally, it occurs in your hometown, and it can occur literally next door.” Others, for example, Claire, also a social worker, argued that there were no clear indicators of risk, and no demographic profile of a sex trafficked person who “could look like your mother, for heaven sakes!”

**Table 1:** Theme: Foundational knowledge (with representative quotes)

Code: Definitions of sex trafficking
*Physicians*
John: If we were talking about [education for] any other type of issue that I see in the emergency department or hospital, [start] from the background and definitions, we would then talk about the epidemiology, and classic presentation of that condition. […] [and] background that [includes] the epidemiology and the sort of typical demographics.
Miss Spell: Education and awareness around what is [sex trafficking]?
*Social workers*
Claire: [Training should explain] what’s sex trafficking like? What? Why, what’s going on? I don’t even know and maybe I haven’t even heard of this.
Ruby: [Education should include] definitions.

Code: Prevalence of sex trafficking
*Physicians*
John: In developing an approach to this situation, the starting point would be the background of how pervasive this issue is in Canada and what are the areas where there might be higher activity.
*Nurses*
Betsy Peacock: I think it, my sense is, at least from the short time that I was at that clinic, is that this is an issue that is much bigger than any of us recognize. And particularly from a domestic standpoint which I don’t think people think about. I know I didn’t before working there.
*Social workers*
Ruby: People need to know that human trafficking is a problem in Canada. You often think it happens across the border or in different countries, but it’s happening here, right in our homeland.
Claire: I think it’s because we’re not aware of how prevalent it [sex trafficking] is and what kind of people are involved, how widespread it really is. I think that makes us all ignorant and absolutely just like, ‘Oh right, how often is it really happening?’
Claire: They could be living at home and being trafficked too. I really think it’s crucial for everybody to understand that. You know the prevalence and how to work, what to look for, and what it looks like. I really think it’s crucial for everybody to understand the prevalence [of sex trafficking].
Jane: I think when we talk about childhood abuse, or we talk about the impact of divorce, we also need to be talking about the prevalence and the impact of trafficking and that it [sex trafficking] occurs, it occurs locally, it occurs in your hometown, and it can occur literally next door.

Code: Risk factors
*Physicians*
Rachel: In our region or in our area, it’s always nice to know what are the current data? Who are the more vulnerable people? Who are these groups? Because I don’t know from a data perspective. What I know is just from news articles and things like that. But it would be nice to know what are some of the factors that we need to be on the lookout for.
John: In developing an approach to this situation […], it might look like defining the communities more likely to be affected. The type of gender or sort of the genders that you might expect to see over-represented […], an image of the sort of classic presentation but then also highlighting other demographic [groups] that are affected.
Sandra Smith: I think [we need] information about the particular demographics of those that are at risk and, you know, things to look out for.
*Nurses*
Vinnie: We have professional development days. So, if there was a course offered, we could take it. And I would be interested to be honest, if there were such a course. […] [For example], I’m uncomfortable even when someone tells me their suicidal. I still don’t know what to do.
*Social workers*
Ruby: [We need to know] What makes people vulnerable?
Corinne: But it’s clear as mud. […] It’s easier for someone who’s quickly seeing a patient in the hospital to be like, ‘Oh, you’ve run away from home’, or ‘Oh, you meet this category’, and these are the possible risks for this individual versus someone who might be seen as stable.

Code: Recruitment processes
*Physicians*
Rachel: But I think it’s always helpful to know what is actually happening in the community. How are people being exposed to trafficking? How are they being recruited?
*Social workers*
Ruby: It probably also [happens on] social media, looking at how people can get recruited from social media, especially children and youth because sometimes that [recruitment] is earlier.
Jane: I think we also need to be talking about [… ][how sex trafficking] occurs online too. […] I think people need to understand that with the online world, it’s […] very different now, just like everything else, it’s moving online and virtually.
Claire: I think there’s a way of recruiting through social media that can often happen. There’s a way that some of the new technology, I think, can pull people in. There’s a vulnerability with some of these pictures and images and the way that sex trafficking can happen online and in the underground Internet; just the transmission of images and information through online means that I don’t know as much about.

Code: Myths and biases
*Physicians*
Rachel: Because I just feel like it could be anybody, there’s no face of this in what you’re looking for… And that’s where I felt some data would be helpful in terms of knowing, as a country, as a province, as the [city named], what are we seeing in terms of people that we are working with, or the people on the ground, the community agencies that maybe are working with people that are sex trafficked. I think, just from a data perspective, it’s also helpful to challenge our assumptions.
Rachel: I think for the vast majority of family doctors or community physicians, it’s just not top of mind for most of us […]. I don’t think most of us would know what we’re looking for, and I think maybe we have the stereotypical kind of situation in our head of people being captured from other countries.
John: Talking about the physical exam, [what are the] sort of signs that we might expect to see if someone is being trafficked, being careful as some of these things might tend towards stereotypes, but like signs of physical abuse or signs of substance use.
Pella: I think part of it is really recognizing our own biases around who is [sex] trafficked and who is not, because we could be seeing people that we don’t realize [are trafficked] in our own practices. Making us open our eyes to that.
*Nurses*
Hailey: ‘[Y]ou [the victim] objectively sound like you’re reciting the plot of a film to me.’ […] Even knowing that that’s what human trafficking looks like, I still have to fight down that impulse of like, they are but absolutely putting me on right now. So, somebody who has no experience with that, they’re going to listen to somebody say those things and think, ‘Okay, cool, so they’re high. They’re psychotic.’
Betsy Peacock: This is an issue that is much bigger than any of us recognize. And particularly from a domestic standpoint, which I don’t think people think about. I know I didn’t before working at [named clinic]. I thought of it [sex trafficking as] being children being snatched from parts of Europe and then moved to other parts of the world.
Sarah: [H]ow to check your biases.
*Social workers*
Claire: **[**Health care providers] also have to know what a predator is. And a predator is not just a guy with a bunch of gold rings who’s driving a Ferrari or something and looking at young girls. A predator […] can [also] be professors at universities or whatever and be involved in sex trafficking, right?
Claire: Everybody has to also have an idea that this is just not one group of people that you think it is […]; you have to think outside the box and stop thinking in terms of narrow groups of people [as at risk].
Claire: It doesn’t happen to her. She’s really well dressed. It doesn’t happen to her. She’s got a degree […] or ‘she’s this or she’s that’, or ‘he’s this or he’s that.’ I mean, we make these assumptions about the type of people that things happen to, and you know what they say, of course, about assumptions.
Claire: I really think it’s crucial for everybody to understand […] what [sex trafficking looks like]. And it looks like everybody; it could look like your mother, for heaven sakes!
Jane: I think there is still a misconception that sex trafficking and prostitution almost go hand in hand and prostitution looks like someone on the street corner. I think there is still, for a lot of people, that belief.

Code: Red flags and warning bells
*Physicians*
Rachel: We had a couple of situations where we had people where we couldn’t tell whether they might be in a situation. [[I]it wasn’t something that they [the patient] brought forward to us outright, but it was something where red flags were going up […] where we were concerned. […] It’s not something I would say, ‘is this something we could rule out?’ But not something that we were definitely suspecting and this was definitely happening. It was just that we couldn’t, we just weren’t sure, but it was definitely on a possibility list. And that’s where that sparked some of the conversation that we had about - ‘Hey, you know we really should do some sort of training about this.’ Who knows? Maybe we’re just missing people and not asking the right questions to be able to identify people who may be in a trafficking situation or where they are being groomed or whatever it may be. […] We have in our mind […] what would be the factors that would send off warning bells.
Rachel: That’s why I thought it would be helpful for us just to know and get training, because I don’t know what I’d be looking for. And, it’s just gut feelings that sometimes you get when you’re having a conversation with people that something’s not quite right. But I think that’s the challenge, because you just won’t know if you’re not asking the right questions.
John: [A curriculum should] talk about when taking the history from a patient, sort of the red flag statements that we might expect to hear that would alert us that someone is being trafficked and ways to probe that further. […] On the physical exam, what signs we might expect to see if someone’s being trafficked, being careful that some of these things might tend towards stereotypes, but signs like physical abuse or signs of substance use. […] So, some of the key elements that might come up on [taking a patient history that] that suggest someone is being trafficked and then any additional signs on physical exam that would make us want to probe further if we haven’t already discussed it, if someone is being trafficked.
Sandra Smith: I would say that informally I try to teach the residents and students that I work with, ‘listen to your gut. If something doesn’t seem right, probe a little deeper.’ Kind of just under the umbrella of that, to just be more cognizant [of potential sex trafficking] and the kind of red flags to look out for […] but I think more training on gender-based violence, sex trafficking in particular [is needed] […], and how to approach patients when you’re suspicious, how to approach [a sex trafficked] confirmed patient. You know, other things to screen and look for […] things to kind of look out for, that we would call a red flag. The things to keep in the back of your mind that if you see, [you] should be thinking about someone who’s being trafficked, or if you see something that might be a red flag.
Leslie: I mean, I guess it would be helpful to know, first of all, when to suspect it [sex trafficking]. I can imagine that it’s similar to any other form of child abuse or violence against women, where you’re looking for signs of undifferentiated symptoms, or signs of violence that are inconsistent with the injuries, or STIs and people who are too young, or [making] frequent visits to the doctor, or no visits to the doctor. So, I imagine a lot of those are similar to other things that we screen for.
Pella: I think [we need] clear questions to ask, like in a screening tool. If the screening tool is positive, then what are the next steps? Because the point of having a screening tool is to have something as a next step and it might be variable depending on where each of us works, right?
Miss Spell: How to spot [sex trafficking]? How to ask questions? How to screen, and then, of course, what to do, what to do.
*Nurses*
Sarah: How to even recognize red flags and what to do if you suspect [sex trafficking]. You can create a risk assessment checklist for somebody that you think might be trafficked. There are definitely signs that you’re going to see.
Mako: I guess things to watch, signs to watch out for that are less obvious or even questions to ask, […] signs of violence, or they come in with a lot of bruises, or things that prompt people to investigate a bit more. […] I guess I’m thinking of signs that are not necessarily physical or things that are harder to immediately notice.
Vinnie: Recognizing the signs and symptoms […] in all ages and populations, with or without a[nother] person there, because it might be different, they [the patient] might act a little differently then. What are the questions you would ask to know if someone is being trafficked?
Betty: I would imagine they would feel and would appear quite withdrawn, perhaps avoiding eye contact, giving indirect answers, not engaging, having very blunted-affect. Physical concerns too, I would imagine. I mean I truly don’t know. I feel I need more education, but this is just my basic knowledge. I’m not saying this is right, but these are my guesses.
Betsy Peacock: I suspect I do see [sex trafficked individuals] on a regular basis. What I don’t have are good tools on how to assess that and I would love that. One of the things that interested me in doing this study is the idea that maybe at some point there could be tools that health care providers could use to screen their patients in a sensitive way. Because I do work with a lot of women who are in the sex trade and I wonder how many of them are doing it [being sex trafficked]. […] I think we see a lot of [sex trafficking] here, and we don’t recognize it and we don’t use specialized tools to not only screen people, but to understand what to do if we find out that’s the case. What resources do we have to help them, if they’re ready to leave a bad situation.
Sharon: I think in health care in particular that’s tricky because it’s not like you can get a survey of people who have survived and it’s not like there’s a right way to ask it, or there’s someone who comes in with the flag that identifies that they’re [a] survivor of sex trafficking. So, we have a lot of work to do, right? […] And what if they can’t [tell you they’re being sex trafficked]? I’ve had people about whom I was very suspicious that this is happening to, but they can’t tell you that.
*Social workers*
Lisa: I would want to learn the warning signs; what are things I should be looking for?
Ruby: [We need to know] the warning signs.
Bugambilia: There’s a lack of knowledge and information for frontline workers. If people don’t have the information on how to understand the vulnerabilities, the presentation [of sex trafficking]… If you don’t have the basic knowledge, unless the client disclosed [sex trafficking] to you, you won’t know [they’ve been sex trafficked].
Corinne: From my nursing colleagues, when they went to a conference, they were like, ‘wow, that’s really sad.’[…] And then it just falls off in the wayside, right? […] How do I identify [sex trafficking]? I think that’s still a hard piece because it is so tricky. And I think we’re still trying to understand how to identify someone that’s being trafficked because there’s a huge spectrum. […] But I think having more training on how to identify [sex trafficked persons. […] I think that’s the big part, to have more training on identification and the topic of ensuring that if I’m a nurse or doctor, then how do I [manage] those follow-ups?

Several participants identified the influence of myths and biases on their understanding of domestic sex trafficking. For example, Rachel, a physician, said “I think maybe we have the stereotypical kind of situation in our head of people being captured from other countries.” The same sentiment was expressed by a nurse who shared, “This is an issue that is much bigger than any of us recognize. And particularly from a domestic standpoint, which I don’t think people think about. I know I didn’t before working at [named clinic]. I thought of it [sex trafficking as] being children being snatched from parts of Europe and then moved to other parts of the world” (Betsy Peacock). Ruby, a social worker, was adamant in stating, “People need to know that human trafficking is a problem in Canada. You often think it happens across the border or in different countries, but it’s happening here, right in our homeland.”

In the absence of evidence-based knowledge about domestic sex trafficking and the contextual variables that may contribute to a person’s risk of being trafficked, such unconscious myths and biases proliferate and can influence providers’ practices. Pella, a physician, observed, “I think part of it is really recognizing our own biases around who is [sex] trafficked and who is not, because we could be seeing people that we don’t realize [are trafficked] in our own practices.” Similar concerns preoccupied Claire, a social worker, who noted that most health care providers are largely unaware of the issue of domestic sex trafficking and have misconceptions about how often it occurs and to whom. She imagines them saying, “Oh right, how often is it really happening?” and thinking “It doesn’t happen to her. She’s really well dressed. It doesn’t happen to her. She’s got a degree […] or ‘she’s this’ or ‘she’s that’, or ‘he’s this’ or ‘he’s that.’ I mean, we make these assumptions about the type of people that things happen to.” Sarah, a nurse, wanted to know “how to check your biases.”

Some participants were clear about their need to also learn more about the various recruitment tactics, including newer online and social media outlets, being used by traffickers to lure potential victims. Hailey, a nurse, indicated how some of these tactics result in scenarios that not only challenge any preconceptions one might hold, but also sometimes even strain belief:

[Y]ou [the victim] objectively sound like you’re reciting the plot of a film to me. […] Even knowing that that’s what human trafficking looks like, I still have to fight down that impulse of like, they are absolutely putting me on right now. So, somebody who has no experience with that, they’re gonna listen to somebody say those things and think, ‘Okay, cool, so they’re high. They’re psychotic.'

Claire added that in addition to knowing how to recognize a sex trafficked victim, health care providers also need to learn about potential traffickers:

And a predator is not just a guy with a bunch of gold rings who’s driving a Ferrari or something and looking at young girls. A predator […] can [also] be professors at universities or whatever and be involved in sex trafficking, right?

Vinnie, a nurse, suggested, “[W]e have professional development days. So, if there was a course offered, we could take it. And I would be interested to be honest, if there were such a course” as he would then have more confidence working with sex trafficked victims. Corinne, a social worker, argued that there are no effective shortcuts to learning about sex trafficking, saying:

I think that’s still a hard piece because it is so tricky. And I think we’re still trying to understand how to identify someone that’s being trafficked because there’s a huge spectrum. […] But I think having more training on how to identify [sex trafficked persons. […] I think that’s the big part, to have more training on identification.

### Navigating the encounter

As the diverse health care providers considered the steps they would take to care for a domestically sex trafficked patient, they acknowledged challenges in the care encounter related to their lack of knowledge, training, and experience (see Table 2). Participants made explicit references to needed training on opening the conversation and broaching the subject; providing an appropriate response; as well as whether and how to record, report, or document the exchange.

**Table 2:** Theme: Navigating the encounter (with representative quotes)

Code: Opening moves
*Physicians*
Sandra Smith: If you can help provide a good approach to broach the subject in kind of a non-confrontational way, and in a way that’s just to show that ‘we’re here to help but you can always come back, if you don’t feel ready to talk about it today, you can come back next time. We’re always here to listen.’ I think just having an approach and the right language to use would be really helpful. I think it would be really important to know how to approach patients you’re suspicious [may be sex trafficked], how to approach a confirmed [sex trafficked] patient.
*Nurses*
Betsy Peacock: I guess maybe even knowing who to screen or how to sensitively broach that topic with an individual who you’re concerned about.
*Social workers*
Lisa: Being mindful of the approach, what approach [you] would use. So, working in anxiety and depression, it’s a lot about challenging, ‘Why don’t we get out of bed? Why don’t we chat?’ But I would imagine in [sex trafficking], the approach wouldn’t necessarily be about challenging, but maybe a different approach. So, what would be the therapeutic approach when working with [sex trafficked persons]?

Code: Asking questions
*Physicians*
Pella: I think part of it is […] having a practical tool, a set of, these are the questions to ask that have high yield on getting answers around like, […] ‘Are you being trafficked or not?’ Maybe there’s a way of asking that. […] If we want to just ask every patient that we see these questions, we have to find ways of doing it that normalizes that we are asking these questions. So, a bit of a script that might feel a bit unfamiliar at first, but really a nice way of asking those questions that will elicit responses. Or at least maybe not the first time around, but maybe the second time that someone comes in to see us they’ll know that they could maybe disclose, because we ask, we have a broad level of understanding. Because we’re simply not asking it now means that we don’t [have that broader understanding] and then it’s harder for patients to want to bring that forward. I think [we need] clear questions to ask.
Miss Spell: Education and awareness around […] [h]ow to ask questions.
Leslie: But something else that would be worthwhile learning is […] how to ask the question. You know we learn a lot about asking questions around domestic violence and that sort of thing, but I suspect these questions would be slightly different.
*Nurses*
Vinnie: The questions to ask if […] let’s just say that they are with another person present. How to get that person to leave the room where it doesn’t make it seem like they are doing something intentional. How to ask those types of questions. And just say, how to get them out of the room. And what are the questions you would ask to know if someone is being trafficked and what are the resources available?
Mako: I guess […] even like questions to ask […]. [I would like] even examples of leading questions, if you suspect someone’s been trafficked, instead of sort of bumbling in the dark.
San: How do you interact with certain populations being trafficked? How do you address [it] with Indigenous women? How do you interact with the Black community? How do you interact with the Asian community? […] Just because if someone is being trafficked, it doesn’t mean that you can use the same toolkit for each person.
*Social workers*
Claire: I think [health care providers] need to first of all to know what questions to ask,
Jane: I think having really practical training on what to say and what not to say would be important.

Code: Trauma-informed care and safe spaces
*Physicians*
David: We don’t have any formal training around it [trauma] but the organization now has seen it as a priority area that we need to build people’s capacity around. Trauma training is happening for social workers and clinicians, but we need training around this as well.
Helen: When I did my presentation, people were saying, ‘but how do you specifically say it? You’re telling us like, [to be] trauma-informed. And you’re telling us, make it a safe space for disclosure. But truly, how do you do that? What words do you use and how do you use them anyway?’
John: How to navigate the interview or interaction, when the patient’s accompanied by someone who might be trafficking them.
Rachel: What do we do? How can we support people if we’re worried about someone? What resources can we connect them with and how can we best support them just to let them know that we’re here for them. Because I think you don’t want to isolate these people, and you want them to know where they can turn for help when they’re ready to reach for help.
*Nurses*
Vinnie: Let’s just say that they are with another person present. How to get that person to leave the room where it doesn’t make it seem like [we] are doing something intentional. [How] to ask those type of questions. […] How to get them out of the room? […] So, if it was a child, what if a person wants to be around them all the time because they want to be able to control the scenario, how would you get that older person out? Like what would be some of the tactics or techniques to say or do to get that person to leave the room even for a few minutes?
Sarah: Still work with compassion and caring with these patients; not just treating their physical illness but treating them as a whole person.
Sharon: I work in an emergency room, so I see people very temporarily. But how do I create a therapeutic relationship with them in 20 min? Can I? I can’t often. Who does then and what is their [the patient’s] safety? That’s really tricky to do in people who don’t ever feel safe and don’t want to be attached to anything, because that in itself is an essence of unsafety.
*Social workers*
Ruby: A lot of maybe therapists or physicians don’t ask about people’s trauma histories. They don’t have the trauma-informed care lens, so I think that would stand in the way [of asking about sex trafficking]. I guess a lack of knowledge about gendered violence, about this type of violence, would stand in in the way. People being afraid if they ask you might open a can of worms and not know how to deal with the client if they dissociate, or if they come up emotional, or if they have a flashback in their office. I’m just hypothetically thinking what might happen, what might happen that stands in the way, or not knowing what resources [are available], how to help, where to begin to help the person.
Ruby: I think that if the research suggests it, I think then that it makes sense to develop [a] trauma sensitive protocol for people who experience human trafficking. For example, people who are trans folk may need something different than someone who identifies as a woman who’s been abused. It’s a different experience.
Jane: But it’s important for some of those teachings to speak about the mental health impact as well. You know, we can provide all the support to a person, but if they’re struggling with a trauma response or hypervigilance or fear, that needs to be addressed as well.
Corinne: If there’s more training on mental health for primary care, and how to approach individuals, then I think that lens would shift a little bit less. But if you’re coming from a really medical viewpoint, you might miss a lot. And I think we are shifting. I think that we are changing because of our awareness of mental health. Because that’s going to be the presenting thing; what’s going to come out is mental health, sort of the symptoms of what’s happening. But, whether the abuse is trafficking, or anything else, it’s going to come out through that [mental health symptoms]. So, I think if there’s more training on how to support [people] and a lot of, some of the practitioners are really uncomfortable because they want to come in, to treat, and try that physical support. […] So, they might feel out of their capacity about how to [provide mental health] support.
Claire: I think it’s super important that every single woman or male, because they are trafficked as well, when they come to you seeking [care], it does not matter what it is, that it is okay to ask about any anything in their life that [is] violence [related] or any sex that was non-consensual […] to let them know that this is a safe place, and if there’s anything to disclose or whatever, that this is an opportunity to do that. ‘There’s no police involvement in this relationship that we’re having right now, whatever it is.’ If they want to remain anonymous or whatever, but that there’s some kind of opening somewhere where someone can disclose, if they want to, that they understand that it’s a safe place to do so.
Zack: Because of the role that I function in is as a mental health therapist doing counselling psychotherapy. So, through this current role that I’m in, for me, specifically probably being able to talk about power out or training around power dynamics specifically through the lens of sex trafficking, sex work more broadly, and strategies on navigating resistance to change through that same lens.

Code: Immediate next steps
*Physicians*
Pella: I think the big one is that we often don’t know what to do after we ask and so, we don’t ask. If we don’t have an option or a set of resources to share with people after we find something out, in medicine generally, we don’t order any tests unless there’s something that we can do about those tests. And so, we don’t ask questions if we can’t help by asking. I think we do have to shift some of that thinking to asking the question may open the discussion around there are things that we can always do, like in terms of maintaining safety, and maybe [its] long acting contraception if they don’t wanna get pregnant or, if that’s not an option, if they’re having unprotected sex, maybe that’s something we can provide that’s like a hidden form of contraception. Like there’s other things that we can do, but I do think it’s […] a big constraint […] not having next steps.
John: I would want to know about next steps, like the supports to potentially contact medical legally [such as], what I should be aware of for pediatric populations. I haven’t done pediatrics in like a long time now. But there’s more mandatory reporting standards for people who are below the age of 18. So, for minors, what are my medical legal obligations? But then, even for the adult population, like what? What would be constituted mandatory reporting more commonly for our age group? […] How does the situation change if there is a minor involved as the child of the person being trafficked?
Rachel: I think it’d be helpful to know […] in conversation with people who have been trafficked or who are in those situations, what did they want from their health care provider in terms of resources, in terms of support?
*Nurses*
Sam: I think really getting more education about how to really help a person exit and what are the resources available for them on exiting.
Betsy Peacock: We don’t [have] specialized tools to not only screen people, but to understand what to do if we find out that’s the case, what resources we have to help them if they’re ready to leave a bad situation. […] And that’s something I would like if (I should say, when) we finally do the training. That we have a sort of action plan if you identify it, more than just, you know, telling somebody to go to the police.
Betty: What would make it better is somehow having a plan for how you’re going to handle that person waiting outside in the car. Which I don’t know what the good plan would be for that. That’s where I feel like I need more education, more support to know how you logistically do that if they’re even, if they’re ready to leave right now. Like, I don’t know how you would logistically do that other than calling the police. But even that could have further […], even if they’re calling the police and get backup, I’m like wait! It’s way more complicated than that. And even with police imminently and woman shelters imminently involved, I feel that there could be still further downfall for the patient. It would not be all roses after that. And so, that’s where I just logistically feel I would need more support as a health care provider, because I don’t know how to do that.
Betty: Hopefully, like I said, what’s missing with my own knowledge is just logistically, how to deal, how to make an imminent safety plan if the trafficker […], if they [the patient] are literally ready to leave now, and the trafficker is outside. But hopefully, if you call these people, they could help you even in that moment, in the crisis [they would] help you, but I don’t know because I’ve never done that.
Megan: Like I didn’t even know something as simple as Victim Services. I didn’t know anything that they could offer until I started my [named hospital] job. I had no idea I could call them for certain situations to help me with, like finding a phone. I didn’t know that until I started this job. So, the mass population of health care workers, they don’t know that they have this. There’s also like a lot of resources we don’t know things until we’re in this niche group and can use them. I still don’t know, even like half my community resources in my own community because I just, I don’t use them or don’t need them for my job. So, there’s like a lot of stuff that I just don’t know well enough to use it.
*Social workers*
Bugambilia: I’d rather learn from women or from men who have lived the experience, have come out of it, and they can tell me, ‘This is the best way a social worker or you a client advocate or peer support worker has supported me.’[…] But for me, I would like to know as a social worker, ‘How can I best walk with you through this so you can come out to the other side you know, if that’s your choice,’ because it’s a choice.
Corinne: If I was a nurse or doctor, then how do I have follow-ups [with sex trafficked persons]? […] I think we’ve tried, with the promise of ‘okay, what’s the next step?’ […]; especially if that’s not in the physiotherapist [’s scope] or that’s not in my scope. How do I approach that relationship when the social worker doesn’t?
Ruby: [We need to know is] how to create safety plans with people […] looking at what resources are available to help, what shelter systems, what housing options are available once people decide to leave. I don’t know. People decide to make a report to the police, [then] how victim services can be of help. Also, getting a doctor if people are, you know, abusing substances or have having sex with who knows who, to make sure that they are okay from a medical standpoint. And then, you know, later down the road, like employment opportunities, building a life, rebuilding a life, or building a life. Trauma therapy. Yeah. So, all of that, I think, should be included [in training].
Lisa: And […] if I do suspect [sex trafficking], What are the next steps? Is it providing them the housing options? Is it providing them with a shelter? So, what to do afterwards [after identifying sex trafficking]?
Claire: Everybody needs their resources, you know. They need, all the health care providers need, to be trained and walked through all the resources and everything that’s out there for them, for their clients, their patients, for whatever they, what’s available, and how quickly if it’s available.

Many physicians explicitly spoke of wanting a set of questions or a standardized tool to help them screen patients about potential experiences of sex trafficking. As Pella said, “We have to find ways of doing it that normalizes [it]. So, a bit of a script that might feel a bit unfamiliar at first, but really a nice way of asking those questions that will elicit responses.” Mako, a nurse, and social workers, Claire and Jane, also wanted to learn the questions that would facilitate disclosure. Mako asked for “examples of leading questions, if you suspect someone’s been trafficked, instead of sort of bumbling in the dark.” Jane, a social worker, agreed saying that she thought it important to ask questions and to ask them in supportive ways but equally important to learn what not to say, while San, a nurse, argued against a single tool, saying that what was needed were approaches that could be tailored to diverse patient populations. As San put it, “How do you interact with certain populations being trafficked? How do you address [it] with Indigenous women? How do you interact with the Black community? How do you interact with the Asian community? […] [I]t doesn’t mean that you can take the same toolkit for each person.”

There was recognition across providers that even with an appropriate tool, specific questions to ask, or words to use, they also needed to know how to establish safe circumstances for sex trafficked patients to feel comfortable enough to disclose. Helen, a physician, noted “[Y]ou’re telling us, make it a safe space for disclosure. But truly, how do you do that? What words do you use and how do you use them anyway?" Vinnie, a nurse, also had concerns about the potential trafficker accompanying the sex trafficked patient. He wanted to know, “How to get that person to leave the room […]. How to ask those types of questions. […] What would be some of the tactics or techniques to say or do to get that person to leave the room even for a few minutes?”

For some, considerations of safety included understanding the impacts of trauma on individuals and how to practice in a trauma-informed way. David, a physician, noted that “We don’t have any formal training around it [trauma] but the organization now has seen it as a priority area that we need to build people’s capacity around. Trauma training is happening for social workers and clinicians, but we need training around this as well.” Ruby, Corinne, and Jane, social workers, identified the general lack of education or training about trauma, its impacts on individuals, and how to provide trauma-informed care, as barriers to engaging patients in conversations about sex trafficking. For Bugumbilia, a social worker, any training should focus on learning from those “who have lived the experience”:

I’d rather learn from women or from men […] [who] have come out of it, and they can tell me, ‘This is the best way a social worker or you a client advocate or peer support worker has supported me.’[…] But for me, I would like to know as a social worker, ‘How can I best walk with you through this so you can come out to the other side you know, if that’s your choice,’ because it’s a choice.

### It just seems so much bigger than Me”

The question about what to do once the proverbial “can of worms” had been opened (after questions about domestic sex trafficking were posed and a positive response elicited), preoccupied many of the participants and sometimes left them feeling defeated (see Table 3). They worried that acquiring knowledge about domestic sex trafficking was not enough, that training on its own would not sufficiently equip them with what was needed to help their patients. Corinne, a social worker said:

**Table 3:** Theme: “It just seems so much bigger than me” (with representative quotes)

Code: Essential supports and services
*Physicians*
John: For example, in the hospital if I was concerned that someone is being trafficked, I would probably pick up the phone and call the social worker, but also our psychiatry colleagues. And even if the psychiatrist isn’t coming to treat acute mental health issues, I guess I’m just hoping that they at least have more experience with navigating the psychosocial dynamic and navigating the legal issues, because it’s something that their specialty might see more commonly.
Bob: And they’re trying to screen people for sex trafficking which I think is good, there’s more awareness. But I think the difficulty is ‘okay, well, you’ve screened it and then if you have a positive screen, then what? And it’s actually much worse because it’s sort of like, ‘Oh, now we know about it’. I feel like it’s kind of disappointing for the patients. It’s like, ‘Oh, great! Now you know that I’m being trafficked and you still have nothing meaningful to offer me?’
Miss Spell: [I would want] a trained provider, but trained providers from multiple disciplines. If you do this work, you know you put patients at risk if you don’t have the resources that you need to support them for success. And me, as an individual, not good enough. I’d need to be part of, as I said, connected to legals, to housing, to all the psychosocial stuff that would be required for extricating them from the situation safely, and not put them at risk of being murdered, or re-trafficked, or moved, right? So, I wouldn’t dare do this without [a team].
Sandra Smith: I think even more important would be, like, the individual like for [named city], Ontario, which is where I work. What are the resources in this city? What can I do to help someone in this city?
*Nurses*
Sharon: Every community, it’s a bit different, like, but I think people need a roadmap depending on where you are. If someone is willing to say, like, in some capacity, ‘maybe this happened to me’, what does that look like in your area?
Sarah: Having somebody as a main resource to be able to go to would be great. I feel like a social worker would be optimal for starting all of that. But again, when I had touched base with social workers, they had no idea [about sex trafficking]. And that’s kind of sad because I feel like it should just be something that’s built into our training. That it shouldn’t be that when it happens, you’re left trying to figure out what your resources are. I feel like the resources should be already put out in an e-learning module.
*Social workers*
Ruby: What are [sex trafficked people] struggling with? Specifically, what are we using in terms of modalities and practice that can be transferred to that population? And what do we need to learn specific to that population? Where did these people go for help? Is there groups for them that they can like access mental health support? Is there shelter specific to help them transition from sex trafficking life to safe harbor? How to get them set up financially, like getting ODSP, or Ontario Works, or finding jobs, health care? Do they have a GP? You need a GP to get a referral to this program. If you don’t have a GP, you know, you’re at a disadvantage. Not everybody has a GP. And do they have, is their medical care being taken care of? Yeah, that’s a big one.
Bugambilia: I would like to know what is available systems-wise on how we can support women. What is available in terms of resources outside of what I already know?

Code: Individual and system capacity
*Physicians*
Pella: Can I call any colleague? It could be a dietitian who has an interest in this, or anybody who might be interested in it? But they are also really strapped for time right now. So, it’s just building in the resources and knowledge across the board. […] It really has to be not just training directed towards clinicians, but to everyone who’s on your health care team. Because then, it again creates that culture of change, of support, of non-judgment that lets people feel safe.
Miss Spell: Because people aren’t going to ask anything if they don’t know what to do and we need specialty care in this area. And we may need to change the way we fund health care, or at least a piece of it. You know when you have 15 min to see a patient as a family doc[tor], you’re not going to see this, you’re not going to spot this [sex trafficking]. And [family doctors], they’re our gatekeepers. They see the vast number of, when I say family docs, let’s say primary care, right, they see the vast number of our patients.
Miss Spell: I think recognition [of sex trafficking] and training and being embedded in a system that works for patients who’ve been sex trafficked [is needed]. We need an entire system developed. It’s not good enough to [only] train, because I’m a doctor, so I’ll speak to doctors, it’s not good enough to train doctors about recognition and treatment [of sex trafficking] in the office. There needs to be a dedicated team of professionals to do that [sex trafficking] work […] a program not limited by specialty or niche, right? We can’t do this without police. We can’t do this without legal. We can’t do it without housing. The infrastructure and the training are not there, at least not in medicine, it’s not there. Or if it is there, it’s not adequate.
*Nurses*
Hailey: When are you going to give us that education and how are we going to absorb it with every other thing that we’re having to do right now? The reality of it is, if you tell most nurses that they’re going to have to go through a human trafficking module, they’re just going to click through it while they’re doing something else because they just don’t have the time. […] There was talk about installing an external or an internal educator at our site to talk to inpatient mental health and labor and delivery, and emergency, and talking to all those groups that are likely to encounter people who either are currently trafficked or have been victims of trafficking or sexual assault. But it’s just like I said, it’s difficult to develop those education programs and it’s difficult to implement them because we just don’t have the time or the bandwidth to be absorbing all that new information.
Mako: [We need] staffing, knowledge training. I guess, protocols. Like, I know we talked a lot about bedside manner and trust but I also feel like there should be standardized protocols, so that people don’t fall through the cracks, especially if we’re training new staff, it’s probably more helpful if there’s some sort of flow sheet or easily accessible extensions or people to reach out to. […] Or even like opportunities for feedback from staff and patients and have that reflected in how they change staff and policy and all that. Even like quick […] e-learnings every year.
*Social workers*
Corinne: I think it goes back to, ‘Okay, who do I connect with? Do I call the community health worker on call? […] If I feel it’s out of my scope, do I call 911? I’m referring to a medical fix-it model, what do I do? I think with trafficking, it’s so grey, I feel like it’s hard to put it on a policy, because it’s, then it’s restrictive […]. How do you make that a policy when it’s so, we’re still trying to figure out what this looks like. So, I think that’s some of the areas that came up.
Corinne: Oh, I think it’s very surface level. Hey, we get it. We know the stats. We get it. There’s some identifiers [but] now what? And then it’s especially, if you’re working [in] the helping field, it’s so defeating because we hear these stories and then it’s upsetting, because what can I do? So, there’s a lack of services, there’s a lack of community supports. How do I help? When you walk away from these conferences feeling like ‘well, that’s another problem, I can’t solve.’ And that’s what I heard from some colleagues, just going yeah, ‘that’s great but I’m so depressed. […] It just seems so much bigger than me.’ So, if we have that perception, nothing’s going to change because we just keep saying, ‘well, I just hope it doesn’t happen to one of my nephews, or nieces, or someone I know […].’ So, I think there’s a sense like it’s just too big to solve. And then people just kind of go back to their regular routine.
Bugambilia: I think we have a system that is racist and this is something that many organizations do, they do performative stuff, like they do training on anti-oppression and racism but I don’t think that’s enough. I think organizations need to change from the board. They have to have representation at every level, so people have choices. Because especially in the health care system, the health care system can make people sicker just by the way they are treated. So, I will say that there have to be meaningful changes in the system, and not just the health care system.
Jane: When we think about the long wait list for mental health care understandably, school systems may have someone who they know or may not know is being sex trafficked. But regardless, [they] may have some pretty scary symptomology they’re dealing with. So, what do they do? They refer to the mental health agencies, that makes a lot of sense. But then they’re making referrals and there are long wait lists.

Hey, we get it. We know the stats. We get it. There are some identifiers [but] now what? And then especially, if you’re working [in the helping field], it’s so defeating because we hear these stories and then it’s upsetting because what can I do? […] There’s a lack of services, there’s a lack of community supports. How do I help? […] [W]ell, that’s another problem, I can’t solve. And that’s what I heard from some colleagues […] ‘that’s great but I’m so depressed. […] It just seems so much bigger than me.’

Rachel, a physician, wondered, “What do we do? How can we support people if we’re worried about someone? What resources can we connect them with and how can we best support them just to let them know that we’re here for them?” Pella, also a physician, added, “If we don’t have an option or a set of resources to share with people after we find something out, in medicine generally, we don’t order any tests unless there’s something that we can do about those tests. And so, we don’t ask questions if we can’t help by asking.” Miss Spell, also a physician, noted, “If you do this work, you know you put patients at risk if you don’t have the resources that you need to support them for success. And me, as an individual, not good enough.” Knowing the resources specific to the local community was a point raised by Sandra Smith, a physician, who declared that it was, “[E]ven more important would be, like, the individual, like, for [named city], Ontario, which is where I work. What are the resources in this city? What can I do to help someone in this city?” Sharon, a nurse, concurred:

Every community, it’s a bit different, like, but I think people need a roadmap depending on where you are. If someone is willing to say, like, in some capacity, ‘maybe this happened to me,’ what does that look like in your area?

Participants wished for more direction and suggested resources, as in policies and guidelines. Mako, a nurse, commented:

I know we talked a lot about bedside manner and trust but I also feel like there should be standardized protocols, so that people don’t fall through the cracks, especially if we’re training new staff, it’s probably more helpful if there’s some sort of flow sheet or easily accessible extensions or people to reach out to. I also feel like there should be standardized protocols.

At least one person believed policies would not be helpful, saying, “I think with trafficking, it’s so grey, […] it’s hard to put it in a policy, because it’s, then it’s restrictive […]. How do you make that a policy when […] we’re still trying to figure out what this looks like” (Corrine, social worker). Miss Spell, a physician argued:

We need an entire system developed. It’s not good enough to [only] train, because I’m a doctor, so I’ll speak to doctors, it’s not good enough to train doctors about recognition and treatment [of sex trafficking] in the office. There needs to be a dedicated team of professionals to do that [sex trafficking] work […] a program not limited by specialty or niche, right? We can’t do this without police. We can’t do this without legal. We can’t do it without housing. The infrastructure and the training are not there, at least not in medicine, it’s not there. Or if it is there, it’s not adequate.

## Discussion

The health care providers in this study consistently voiced their need for comprehensive and specialized education tailored to their profession (e.g. social work) and area of practice (e.g. medical, mental health) to combat their sense of inadequacy in caring for sex trafficked patients. The theme, Foundational Knowledge, identified health care providers’ desire for concrete information about sex trafficking and echoed findings from a study by Stoklosa and colleagues in the U.S [31]. Our participants identified needing to learn, for example, the definition of domestic sex trafficking and its prevalence, as well as the various circumstances or situations associated with increased risk of recruitment into sex trafficking, and then what to do with that understanding. In terms of desired training (that is specific skills required), across all of the interviews a salient issue repeatedly emerged: how to identify or recognize a person being sex trafficked, a concern that also emerged from our previous study of social service providers’ identification practices and education needs vis-à-vis domestic sex trafficking [16, 19]. Some of the health care providers referred to needing to better understand the ‘red flags’ or warning bells suggestive of sex trafficking while others wanted a tool or specific set of questions to facilitate disclosure. While the appeal of a standardized protocol to aid health care providers in identifying sex trafficked persons has been recognized as potentially useful [32], our participants, while wishing for something simple to use, simultaneously acknowledged needing the flexibility to tailor or adapt the approach to the diversity of patients encountered.

Despite the challenges associated with identifying potential domestically sex trafficked persons, a health care provider is still one of the most likely initial points of contact with the potential to recognize and assist sex trafficked persons while they are still in the trafficking situation [12]. Health care providers who recognize a patient’s precarious situation, in close consultation with that patient, if desired, can tailor the plan of care to one the patient feels most able to carry out given their circumstances, connect individuals to helpful resources, provide appropriate referrals, and address additional biopsychosocial needs [34] suited to those who remain in or leave the trafficking situation.

Thinking about identifying a sex trafficked person immediately led to other pressing concerns for our participants and these were explored in the theme, Navigating the Encounter. In considering how to open what was considered a sensitive conversation and what to do with positive disclosures, participants voiced concerns focused on various safety-related issues. Their questions and concerns included how to: create a safe space for disclosure, strategize to separate or manage the trafficker when present, provide culturally appropriate and safe ways of discussing sex trafficking, and access other useful resources and referrals [34]. Many acknowledged that understanding trauma and practicing trauma-informed care was a key element in creating a safe encounter for a sex trafficked patient but acknowledged their own lack of knowledge and shortcomings in this area. Although variously defined, trauma-informed care generally refers to a supportive attitude and sensitivity to the potential trauma patients may have/are encountering in their lives and ensuring this understanding is acted upon throughout the organization [35]. For health care providers to be able to effectively help sex trafficked persons, it is essential that they first understand the physical, emotional, and psychological impacts of traumatic experiences on individuals, how to respond appropriately, and ensure that others in the organization also know how to do so. As Chambers and colleagues noted, “Perhaps most pressing is the need for education of health care providers at all levels and in all health care settings on the issue of human trafficking and trauma-informed care for its victims.” [36 (p.350)] Enhancing health care providers’ abilities to recognize, respond, provide trauma-informed care, and appropriately refer to other needed and vetted resources is critical to safely and effectively assisting sex trafficked individuals and ensuring they receive what is most needed, in the most appropriate way, and at the most appropriate time.

Through this study, we learned the extent to which having some education about trafficking, but not enough for providers to feel confident that they knew how to best support a sex trafficked patient, left some health care providers feeling overwhelmed, discouraged, or dejected. Therefore, in designing educational initiatives, one needs to ensure that the content is practical, addresses skills that are appropriate to learners, includes helpful resources and locally relevant referrals. This may be augmented, as we and others have noted, by establishing health system specific protocols focused on multi-disciplinary collaborative practices, integrated organizational support, and established networks of vetted community referrals (e.g. governmental and nongovernmental systems) for patients [9, 14, 17, 23, 37]. When health care providers take on the work of supporting sex trafficked persons, they are at times thrown into a role for which they have had little preparation, education, or organizational support. The theme, “It just seems so much bigger than me” indicates the extent to which participants recognized that systems beyond health care need to become involved to effectively help sex trafficked patients. “How do I help?” was both a question about what a single person could do as well as a veiled cry for the development of institutional commitments to (a) ensuring health care providers receive the education and training they need and (b) increasing staff numbers to levels sufficient to allow providers to spend the necessary time with the sex trafficked patient. It suggests as well, the need for greater investment in developing the full range of collaborations (internal and external to the health care institution including to legal and housing services) required to help a sex trafficked person. As Miss Spell said, “you put patients at risk if you don’t have the resources that you need to support them for success.”

Recently the World Health Organization noted that in addition to health care providers’ lack of knowledge about trafficking and how to identify and care for trafficked individuals, the most significant structural barriers were “understaffing and underfinancing” along with “insufficient resources for specialty services” [38 (p.16)], a finding we noted in our study of social service providers’ challenges in meeting the needs of sex trafficked persons [17], and noted in other studies exploring health care provider challenges in addressing the needs of trafficked persons [17, 23]. Even with access to the very best training materials and all the good will in the world, those working in health care cannot provide all that is needed to help a domestically sex trafficked person be healthy, stay safe, and when desired, successfully exit the trafficking situation. It is clear that other resources and services such as housing, employment, and counselling, are also urgently needed [39]. 

There are some limitations to this study. First, the study was conducted in a single province and while Canada has a universal health care system, there are provincial differences in the organization and financing of health care that may impact providers’ education and training on domestic sex trafficking and the delivery of health care to those who have been sex trafficked. Second, not all regions of the province were adequately represented, which may mean issues prevalent in these regions have not been fully considered. Finally, as in all qualitative research, the findings may not be generalizable to other settings or professions.

## Conclusion

While health care providers clearly lack the specific education and training to recognize and appropriately care for domestically sex trafficked persons and wish to know more about the very fundamentals about sex trafficking through to managing the encounter, they are also keenly aware of the need for other community-based resources, the necessity of having supportive infrastructure, and established connections to other essential services such as legal and housing. Even the best content and most inclusive training can only meet some of a sex trafficked patient’s needs. As Miss Spell recognized, “We need an entire system developed."

## Data Availability

Data supporting the findings of this study are found in the provided tables. Additional data are not available due to the detailed and sensitive information shared during the interview, the small sample size, and consequent possibility of compromising anonymity/individual privacy of participants.

## Abbreviations

2SLGBTQI+: 2 Spirit, Lesbian, Gay, Bisexual, Trans, Queer, or Intersex
